# RNAi-Mediated Knockdown of Acidic Ribosomal Stalk Protein P1 Arrests Egg Development in Adult Female Yellow Fever Mosquitoes, *Aedes aegypti*

**DOI:** 10.3390/insects15020084

**Published:** 2024-01-24

**Authors:** Mahesh Lamsal, Hailey A. Luker, Matthew Pinch, Immo A. Hansen

**Affiliations:** 1 Molecular Vector Physiology Laboratory, Department of Biology, New Mexico State University, Las Cruces, NM 88003, USA; mlamsal@iu.edu (M.L.);; 2 Department of Biology, University of Texas El Paso, El Paso, TX 79968, USA

**Keywords:** P1, GCN1, RNAi-mediated knockdown, *Aedes aegypti*, mosquito reproduction, ribosomal protein, nutrient signaling

## Abstract

**Simple Summary:**

Increasing levels of insecticide resistance in disease-transmitting mosquitoes highlight the need for alternative strategies to control vector mosquito populations. RNA interference-based strategies have strong potential to become a major component of integrated vector management. To implement dsRNA-based insecticides, effective target genes need to be identified whose knockdown causes death or sterility in mosquitoes. Here, we show that the ribosomal protein P1 is a promising candidate as a target for mosquito birth control using RNA interference.

**Abstract:**

After taking a blood meal, the fat body of the adult female yellow fever mosquito, *Aedes aegypti*, switches from a previtellogenic state of arrest to an active state of synthesizing large quantities of yolk protein precursors (YPPs) that are crucial for egg development. The synthesis of YPPs is regulated at both the transcriptional and translational levels. Previously, we identified the cytoplasmic protein general control nonderepressible 1 (GCN1) as a part of the translational regulatory pathway for YPP synthesis. In the current study, we used the C-terminal end of GCN1 to screen for protein–protein interactions and identified 60S acidic ribosomal protein P1 (P1). An expression analysis and RNAi-mediated knockdown of P1 was performed to further investigate the role of P1 in mosquito reproduction. We showed that in unfed (absence of a blood meal) adult *A. aegypti* mosquitoes, *P1* was expressed ubiquitously in the mosquito organs and tissues tested. We also showed that the RNAi-mediated knockdown of P1 in unfed adult female mosquitoes resulted in a strong, transient knockdown with observable phenotypic changes in ovary length and egg deposition. Our results suggest that 60S acidic ribosomal protein P1 is necessary for mosquito reproduction and is a promising target for mosquito population control.

## 1. Introduction

In the last few decades, the yellow fever mosquito, *Aedes aegypti*, has become an important model organism for the study of nutrient-sensing and signaling pathways, disease transmission, and cellular metabolism and physiology [1,2,3,4,5]. This is due to *A. aegypti*’s capacity to vector diseases, specialized anatomy that is unique from other model organisms, and relatively quick life cycle. In addition, *A. aegypti* mosquitoes are anautogenous, meaning they require a blood meal to produce a clutch of eggs; as a result, their reproductive and nutrient processes are uniquely easy to study [6,7,8]. The uptake of a blood meal triggers several physiological processes including vitellogenesis. Vitellogenesis, the accumulation of yolk (vitellus) in the developing oocytes, starts with the synthesis of yolk protein precursors (YPPs) [9,10,11]. YPPs are synthesized exclusively by the mosquito’s fat body. After synthesis in the trophocytes of the fat body, YPPs are released into the hemolymph, taken up by the oocytes via receptor-mediated endocytosis, and then cleaved into yolk proteins [12,13,14]. 

**Figure 1:**
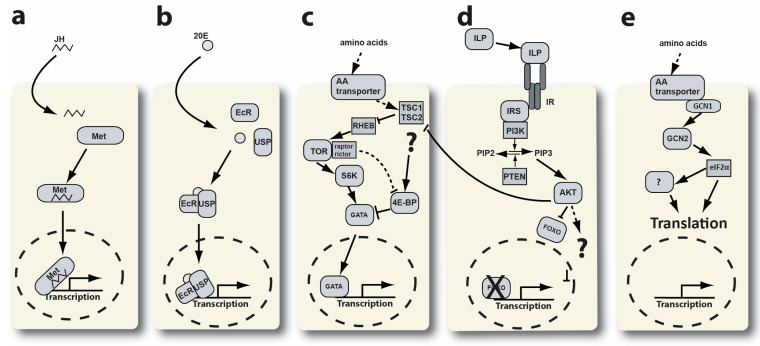
The regulation of vitellogenic gene expression in mosquito fat body cells. This figure is modified from Hansen et al., 2014 [19]. (**a**) Juvenile hormone signaling. JH—juvenile hormone, Met—methoprene receptor. (**b**) Ecdysone signaling. 20E—20-hydroxy-ecdysone, EcR—ecdysone receptor, USP—ultraspiracle. (**c**) TOR signaling. AA—amino acid, TSC—tuberous sclerosis complex, RHEB—RAS homologue enriched in brain, S6K—S6 kinase, 4E-BP—4E—binding protein, GATA—GATA type transcription factor. (**d**) Insulin signaling. ILP—insulin-like peptide, IR—insulin receptor, IRS—insulin receptor substrate, PI3K—PI3 kinase, PTEN, AKT, FOXO—forkhead box transcription factor O. (**e**) GCN signaling. GCN—general control nonderepressible, eIF2α—eukaryotic initiation factor 2 alpha. The dotted circle represents the cell nucleus.

*Regulation of YPP gene expression*. Several cellular signaling pathways have been linked to the regulation of vitellogenic gene expression (see Figure 1). Juvenile hormone (JH), ecdysone, target of rapamycin complex 1 (TORC1), insulin-like peptide, and general control nonderepressible (GCN) signaling pathways have all been shown to regulate YPP transcription and translation in the cells of the fat body [15,16,17,18]. TOR and GCN signaling both involve amino acid (AA) transporters at the top of their respective signaling cascades (see Figure 1c,e). The increase in AAs in a blood-fed mosquito triggers these pathways in the trophocytes of the fat body. This activation is due to the transport of AAs from the hemolymph into the cytoplasm of trophocytes. 

Cationic amino acid transporters. Membrane-bound AA transporter proteins of the SLC7 family transport AAs that are necessary for mosquito vitellogenesis [20,21,22,23]. The AAs histidine, lysine, and arginine are cationic AAs and require specialized transporters called cationic amino acid transporters (CATs) to cross cell membranes. CATs are membrane transporters that facilitate the massive flow of cationic AAs from one mosquito tissue to another [24,25]. The genome of *A. aegypti* encodes five CATs [1]. CAT1, CAT2, and CAT3 are expressed in the fat body of adult *A. aegypti* and the knockdown of these transporters significantly reduces YPP synthesis [20,26], demonstrating the importance of CATs for mosquito vitellogenesis.

Downstream of CATs, TOR, and GAAC signaling regulate mosquito egg development. The abovementioned studies showed that RNAi-mediated knockdown of specific SLC7 transporters reduced AA-induced TOR signaling and overall mosquito egg development [1,26]. Pinch and colleagues further investigated the mechanics and involvement of CATs in nutrient signaling pathways involved in the regulation of vitellogenesis. They performed a yeast two-hybrid (Y2H) screen using the C-terminal end of *A. aegypti* CAT1 and identified general control nonderepressible 1 (GCN1) as an interacting protein. GCN1 is a part of the general amino acid control (GAAC) signaling pathway that regulates the response to amino acid starvation in eukaryotic cells [27,28,29,30,31,32,33,34]. Next, they found that *GCN1* was highly expressed in organs and structures associated with vitellogenesis, with the highest gene expression in the ovaries followed by the fat body. When they performed a knockdown of GCN1 in *A. aegypti*, they found no effect on YPP transcription but a significant reduction in fertility [35]. 

**Figure 2:**
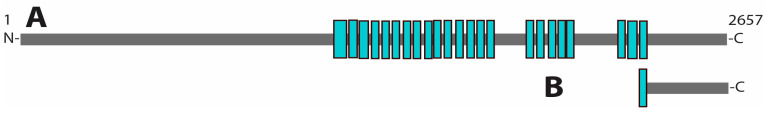
Predicted domain structure of *A. aegypti* GCN1. (**A**) Structure of GCN1 complete protein. (**B**) C-terminal domain of GCN1 that was used for Y2H screening. HEAT domains are shown in blue.

General control nonderepressible 1. GCN1 is a conserved protein in eukaryotic organisms [31,36]. It was first described in yeast as an effector protein of GCN2 in the GAAC pathway. In AA-starved cells, ribosomes collide during translation, creating a disome. GCN1 binds to both ribosomes in this disome and subsequently activates GCN2, starting the GCN signaling pathway [31,37,38]. The N-terminal domain of GCN1 is necessary for robust ribosome binding [39,40,41]. Downstream of this N-terminal domain is a region containing several HEAT repeats, repetitive arrays of short amphiphilic α-helices, that are involved in the formation of protein–protein interactions (see Figure 2A) [42,43]. The C-terminal domain of GCN1 also interacts with colliding ribosomes and mediates the interaction between GCN1 and GCN2 [41,44].

The role of GCN1 in mosquito vitellogenesis has only been recently discovered and there is a significant gap in our knowledge of GCN1’s functions within the mosquito fat body. To fill this gap and uncover novel components in the GCN pathway, we performed a yeast two-hybrid (Y2H) screen using a C-terminal fragment of *A. aegypti* GCN1 (see Figure 2B). In this study, we identified 60S acidic ribosomal protein P1 (P1) as a protein interactor of GCN1 and demonstrated that the knockdown of P1 results in female *A. aegypti* sterility. 

## 2. Materials and Methods

Yeast two-hybrid assay: A yeast two-hybrid (Y2H) screen was performed using the Matchmaker^®^ Gold Yeast Two-Hybrid System (Takara Bio, San Jose, CA, USA Cat. Nos. 630466, 630489, 630498, 630,499 PT4084-1 (071519)), following the manufacturer’s protocol. The GCN1 protein sequence (2656 amino acids) was retrieved from GenBank (accession number: XP_001651826) and a 906-nucleotide C-terminal sequence was amplified using PCR and cloned into a pGBKT7 plasmid. 

This sequence includes a HEAT domain at the C-terminal end:

AELIVIHTRPDPLFVEMQNGIKNTDDSTIRETMLQALRGILTPAGDKMTEPLKKQ IYSMLSGMLGHPEDVTRAAAAGCLGALCRWLNPDQLDDALNSHMLNEDYGDDAA LRHGRTAALFVALKEYPSAIFTDKYEAKICKTIASSLISDKIPVALNGVRAAGYLLQYG MCSDDVKLPQQIIGPFVKSMNHTSNEVKQLLAKTCLYLAKTVPAEKTAPEYLRLVIPM LVNGTKEKNGYVKSNSEIALVYVLRLREGDEVHQKCIALLEPGARDSLSEVVSKVLRK VAMQPVGKEEELDDTILT 

The following primer set was used for PCR amplification of the bait cDNA: 

GCN1 bait forward: 

5′CATGGAGGCCGAATTCGCAGAACTGATCGTAATCCACACC3′ 

GCN1 bait reverse: 

5′GCAGGTCGACGGATCCCGTAAGAATCGTATCGTCTAGTTC3′ 

To clone the GCN1 bait PCR fragments into the pGBKT7 plasmid, we used the In-Fusion^®^ HD Cloning Kit (Takara Bio USA Inc., Orchard Pkwy, San Jose, CA , USA). To transform the bait pGBKT7 plasmid into competent yeast cells (Y2HGold strain), the Yeastmaker™ Yeast Transformation System 2 Kit (Takara Bio USA Inc.) was used. For prey library construction, the yeast transformation protocol from Yeastmaker™ Yeast Transformation System 2 (Takara Bio USA Inc.) was used to prepare competent yeast cells of the prey strain Y187. The Make Your Own “Mate & Plate™” Library System (Takara Bio USA Inc.) was used to construct the two-hybrid library strain, following the manufacturer’s instructions. Library strain construction is described in detail elsewhere [35].

Briefly, whole female mosquitoes, 24 h after a blood meal, were decapitated and their legs and wings were removed. Total RNA was isolated and purified, and mRNA was subsequently enriched using a commercially available purification kit (Takara Bio USA Inc.). cDNA was synthesized using SMART MMLV reverse transcriptase, followed by In-Fusion^®^ cloning into the prey vector pGADT7 AD. Library plasmids were transformed into the yeast strain Y187.

The bait and prey strains were mated, and zygote formation was confirmed using a phase contrast microscope. After screening for yeast colonies that displayed reporter phenotypes, indicating that they contained interacting proteins, individual library plasmids were recovered from these colonies and sent for Sanger sequencing (MCLAB, South San Francisco, CA). DNA sequences were analyzed using NCBI BLASTX.

Mosquito rearing: Five- to seven-day-old female *A. aegypti* mosquitoes (Black Eye Liverpool Strain, BEI resources, Catalog No. NR-48921) were used for all experiments in this study and reared under standard conditions described before [43]. Larvae were reared in pans and fed ad libitum with cat food (Special Kitty, Walmart Stores Inc., Bentonville, AR, USA). Pupae were transferred to Bug Dorm-1 insect rearing cages (30 × 30 × 30 cm; BugDorm, Taichung, Taiwan) and stored in a room with 80% humidity and 28 °C temperature with a 12:12 light:dark cycle. Adult mosquitoes were fed ad libitum on a 20% sucrose solution. 

Protein isolation: Mosquitoes were ice-anesthetized, and their wings and legs were removed. Organs and tissues (head, thorax, midgut, fat bodies, Malpighian tubules, and ovaries) were dissected in *Aedes* physiological saline (APS) [45]. The dissected organs and tissues were transferred into separate Eppendorf tubes with lysis buffer (100 µL IGEPAL, 1 µL protease inhibitor, Thermo Fisher Scientific, Waltham, MA, USA Lot # VA293119) and 1 µL phosphatase inhibitor (Thermo Fisher Scientific Lot # UH288468) and homogenized using a handheld homogenizer (VWR, Avantor; Radnor, PA, USA; Cat. No. 4774-370) with disposable polybutylene terephthalate pestles (VWR, Avantor; Cat. No. 4774-358). The samples were subsequently centrifuged at 12,000 RPM for 10 min at 4 °C. The supernatant was collected in 1.5 mL Eppendorf tubes and protein concentrations were measured using the Pierce™ BCA Protein Assay Kit (Thermo Fisher Scientific Inc.). Protein concentration measurements were performed with a NanoDrop™ 1000 spectrophotometer (Thermo Fisher Scientific Inc.) using the protein BCA program.

Western Blot: Twenty micrograms of protein from each sample was diluted in a 1:1 ratio of Laemmli buffer and 1% beta-mercaptoethanol. The mixture was incubated for 5 min at 95 °C to denature the proteins. Samples were size-separated on a 7.5% acrylamide gel at 100V (Bio-Rad Laboratories, Irvine, CA, USA) for 1 h. The proteins were transferred to a polyvinylidene difluoride (PVDF) membrane using a wet-blotting apparatus and the protocol described in the manufacturer’s manual (Bio-Rad Laboratories). The membrane was then washed four times in five-minute intervals on a rocking table using a wash buffer (TBST + 0.05% Tween-20). The membrane was then blocked with a blocking buffer (3% BSA) for an hour at room temperature with a gentle rocking motion. A diluted GCN1 primary antibody (polyclonal RPLP1 antibody, Catalog # PA5-77002, Thermo Fisher Scientific Inc., 1:500 in blocking buffer) was added to the membrane and allowed to incubate at 4 °C overnight with gentle shaking. The membrane was then washed four times, and a secondary antibody (anti-rabbit secondary Ab, Millipore Sigma, Burlington, MA, USA; 1:1000 dilution) was added and incubated at room temperature for one hour. The membrane was washed again four times. A chemiluminescence assay was performed (WesternSure PREMIUM Chemiluminescent Substrate, Li-Cor, Lincoln, NE, USA). The membrane was analyzed with a C-Digit imager using the Image Studio software v5.5.4. (LI-COR, Lincoln, NB, USA).

RNA isolation: RNA was isolated using the Qiagen RNeasy Kit (Qiagen, Germantown, MD, USA), following the manufacturer’s protocol. For qPCR expression analyses, various numbers of organs/tissues were dissected and combined for each biological replicate in order to isolate at least 1 µg of RNA for each tissue replicate. For the knockdown qPCR experiments, three whole mosquito females were pooled for each of four biological replicates. Wings, legs, and heads were removed. The pools were homogenized in a 1.5 mL Eppendorf tube in 350 µL of lysis buffer (Buffer RLT, Qiagen, Germantown, MD, USA) using a VWR cordless motor (VWR, Avantor; Radnor, PA, USA; Cat. No. 4774-370) with disposable polybutylene terephthalate pestles (VWR, Avantor; Cat. No. 4774-358). RNA concentration was determined using a Nanodrop™ 1000 spectrophotometer (Thermo Fisher Scientific Inc.).

RNAi-mediated gene knockdown: For RNAi-mediated knockdown, double-stranded RNA (dsRNA) targeting *P1* and *green fluorescent protein (GFP)* as a control was synthesized. Primers were designed using PrimerBlast (see Table 1) [46]. To facilitate in vitro dsRNA synthesis, T7 promoter sequences were added to the 5′ ends of both forward and reverse primers. dsRNA was synthesized using the Megascript™ RNAi Kit (Thermo Fisher Scientific Inc., Lot No. 01130674), following the manufacturer’s instructions. Synthesized dsRNA was diluted in APS to a concentration of 1 µg/µL. Sets of *A. aegypti* females (5–7 days post-eclosion) were injected intrathoracically with 1 µL of either the *GFP* dsRNA (control) or *P1* dsRNA (knockdown) [47]. Injected mosquitoes were transferred into Bug Dorm-1 insect rearing cages to recover. Mosquitoes were fed ad libitum on a 20% sucrose solution and stored in the rearing environment described above. Mosquitoes that did not recover from the injections were discarded.

Quantitative reverse transcription PCR (qRT-PCR) analysis: For the qRT-PCR analysis, qRT-PCR primers were designed for *P1* and *β-actin*, a “housekeeping” gene used as a reference. *β-actin* has been validated as a suitable reference gene for qPCR during different life stages and in different tissues of *A. aegypti* [48]. qRT-PCR primers were designed using PrimerBlast (see Table 2). The primer sequences for *β-actin* qRT-PCR primers were previously described. cDNA synthesis and qRT-PCR reactions were performed as described previously [35]. Three repeats were performed, and relative expression levels were either calculated in comparison to whole mosquitoes (for tissue/organ expression analyses) or *GFP*-injected control mosquitoes (for knockdown experiments).

Ovary morphology assay: *GFP*-dsRNA- and *P1*-dsRNA-injected mosquitoes, 24 h post-injection (PI), were fed defibrinated bovine blood (HemoStat Laboratories, Dixon, CA, USA) for one hour. Mosquitoes that did not take a blood meal during this time were subsequently removed from the cages. Prior to the blood meal, 10 mosquitoes were collected from each treatment cage to study the morphology of unfed mosquito ovaries. In total, ten ovaries from each of the four time points (0, 24, 48, and 72 h post-blood meal (PBM)) were collected. All ovaries were imaged using an Olympus CX41 stereo microscope (Olympus Life Science, Waltham, MA, USA). Ovary lengths were determined using ImageJ software Version 1.54d [49]. 

Egg number assay: For this assay, four groups of twenty *GFP*- and four groups of twenty *P1*-dsRNA-injected mosquitoes were blood fed, as described above. A wet filter paper was placed in the treatment and control cages 72 h PBM and collected 96 h later. Egg numbers were counted using a stereomicroscope.

Statistical analysis: All statistical analyses were performed using GraphPad Prism (GraphPad Software, San Diego, CA, USA, Version 9.5.1). A Shapiro–Wilk test was performed to test if the data were normally distributed. An unpaired *t*-test was performed to determine differences in ovary length between the *P1*-dsRNA-injected and *GFP*-dsRNA-injected mosquitoes. An unpaired *t*-test with Welch’s correction was used for each time point to analyze the qRT-PCR data. A simple survival analysis (Kaplan–Meier) was used to make the survival curves. A log-rank (Mantel–Cox) test was used to determine differences between the survival of *GFP-* and *P1*-dsRNA-injected mosquitoes. *p*-values < 0.05 were considered statistically significant for all tests.

## 3. Results

### 3.1. Protein–Protein Interactions of GCN1

The Y2H screen we performed using the C-terminal end of *A. aegypti* GCN1 resulted in the isolation of six separate library plasmids (see Table S1). All six plasmids encoded the C-terminus of the same protein: 60S acidic ribosomal protein P1 (GenBank accession number: XP_001663779).

### 3.2. Gene Expression of P1 in Different Mosquito Organs and Structures

**Figure 3:**
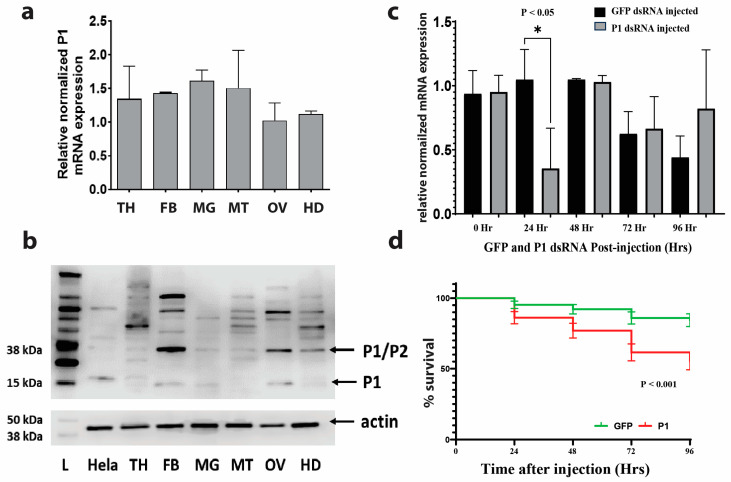
*P1* gene expression and mosquito survival after knockdown. Variation between replicates is displayed as the standard error of the mean (SEM). (**a**) P1 is expressed at uniform levels in various organs/structures of unfed female mosquitoes. Relative expression levels were determined via qRT-PCR (thorax, TH; fat body, FB; midgut, MG; Malpighian tubule, MT; ovary, OV; head, HD). (**b**) Antibody against human P1 detects bands of various sizes in different mosquito tissues. The upper panel shows the results of a Western blot using P1 antibody. The lower panel shows the results of a Western blot using an antibody against actin as a loading control (L, protein molecular weight marker; Hela—HeLa cell protein extract; see Figures S1–S3). (**c**) Transient knockdown of *P1*. Shown are qRT-PCR results of *P1* expression at different time points after injection of dsRNA. The “0 Hr” time point represents uninjected control mosquitoes. *β-actin* gene expression was used to normalize the data. Three pools of three mosquitoes were used for each time point. An unpaired *t*-test with Welch’s correction was used for each time point to determine significant differences in *P1* expression between *GFP*- and *P1*-dsRNA-injected mosquitoes. (**d**) Significant increase in mosquito mortality after *P1* dsRNA injection. Shown is the average proportion of survival for mosquitoes injected with *GFP* (*n* = 192) or *P1* dsRNA (*n* = 196) measured at different time points after injection (see Table S2 and Figure S4 for individual replicate survival curves). A log-rank (Mantel–Cox) test was used to determine differences between the survival of *GFP-* and *P1*-dsRNA-injected mosquitoes.

In unfed, 5–7-day-old adult female *A. aegypti* mosquitoes, we profiled the expression of P1 within six different mosquito organs and tissues: thorax (TH), fat body (FB), midgut (MG), Malpighian tubules (MT), ovary (OV), and head (H). The qRT-PCR analysis revealed a ubiquitous and uniform expression of P1 across the different organs and tissues tested (see Figure 3a). To investigate the protein expression patterns of P1, we performed a Western blot using an antibody against human P1 (see Figure 3b). The predicted molecular weight of *A. aegypti* P1 protein is 15 kDa. A band at this size was observed in fat body, midgut, and ovary samples. Various other nonspecific bands of higher molecular weight were observed in different patterns in different organs/structures. This included a band at 38 kDa, the predicted size of the P1/P2 dimer of *A. aegypti*.

### 3.3. RNAi-Mediated Knockdown of P1 Is Transient

Knockdown efficacy was measured at five different time points post-injection using qRT-PCR. We found a strong knockdown effect in P1-injected mosquitoes 24 h after injection (see Figure 3c).

### 3.4. Mosquito Mortality after RNAi-Mediated P1 Knockdown

Survival was measured over four days after dsRNA injection. P1-dsRNA-injected mosquitoes had significantly (*p* < 0.001) lower survival rates after injection compared to the control (see Figure 3d). Twenty-four hours post-injection, the percentage of survival for GFP-injected mosquitoes and P1-injected mosquitoes was 94.8% and 86.2%, respectively. Forty-eight hours post-injection, there was a 91.3% and 77.6% survival, respectively. At 72 h post-injection, it was 85.2% and 61.8%, respectively, and at 96 h post-injection, it was 83.7% and 56.3%, respectively. 

### 3.5. RNAi-Mediated P1 Knockdown Results in Ovary Phenotype in Mosquitoes

**Figure 4:**
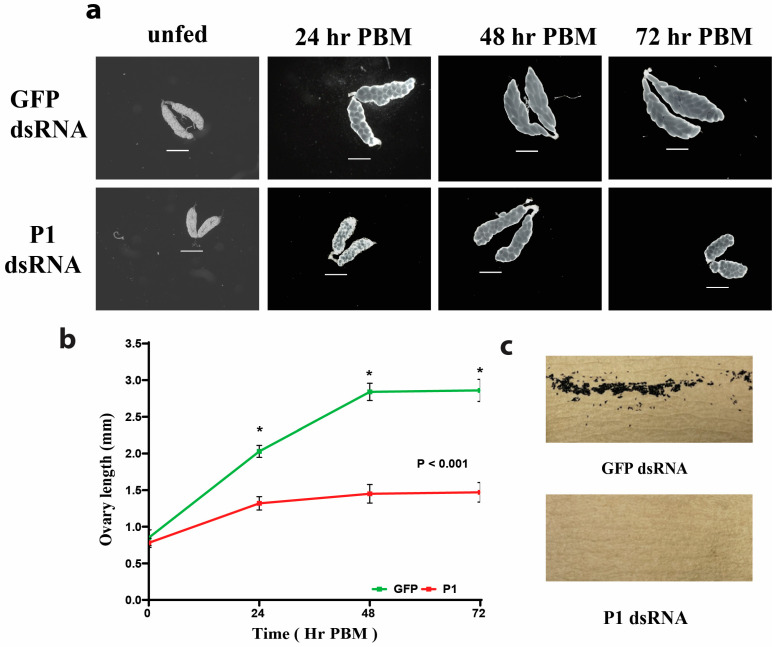
RNAi-mediated *P1* knockdown affects ovary length and egg numbers. (**a**) *P1*-knockdown mosquitoes show a pronounced ovary phenotype. Shown are ovaries from *GFP*- and *P1*-dsRNA-injected mosquitoes at different time points PBM. The top row shows ovaries from *GFP*-dsRNA-injected mosquitoes. The bottom row shows ovaries from *P1*-dsRNA-injected mosquitoes. The scale bar represents 500 µm. (**b**) Ovary lengths at different times PBM are significantly shorter in *P1*-knockdown mosquitoes. Ovary lengths of *GFP-* and *P1*-dsRNA-injected mosquitoes sampled at 0 h PBM (unfed), 24 h PBM, 48 h PBM, and 72 h PBM. The plotted data represent the average length of ovaries (*n* = 10) at each time point. Error bars represent the standard error of the mean (SEM), and an unpaired *t*-test was performed to determine the statistical significance of the difference between groups. (see Table S3) (**c**) RNAi-mediated *P1*-knockdown mosquitoes do not lay eggs after a blood meal. Shown are images of egg deposition papers from *GFP-* (top image) or *P1-* (bottom image) dsRNA-injected mosquitoes. Note the complete absence of eggs produced by *P1*-knockdown mosquitoes. Two replicate trials with 20 mosquitoes for both control and knockdown groups were performed, with consistent results (see Figure S5).

The ovary length of *GFP*- and *P1*-dsRNA-injected mosquitoes was measured at four different time points after a blood meal (see Figure 4). The ovaries of *GFP*-dsRNA-injected mosquitoes had an average length of 0.78 mm before a blood meal. Seventy-two hours after a blood meal, the ovaries had lengthened to an average of 2.86 mm. The ovaries of *P1*-dsRNA-injected mosquitoes had an average length of 0.85 mm before a blood meal. Seventy-two hours after a blood meal, the ovaries had lengthened to an average of 1.47 mm (see Figure 4a,b). The ovary lengths of *P1*-dsRNA-injected mosquitoes were significantly shorter compared to the ovary lengths from *GFP*-injected control mosquitoes at all time points PBM (*p* < 0.001) (see Figure 4b). P1-knockdown mosquitoes did not lay any eggs after a blood meal (see Figure 4c). Egg trays were left in cages for up to nine days (216 h PBM).

## 4. Discussion

Mosquitoes are medically important vectors of diseases like dengue, chikungunya, yellow fever, and malaria [50,51,52,53,54]. Mosquito population control is an effective strategy used to mitigate the transmission of these diseases [55]. Disease transmission is strongly correlated with the reproductive life cycle of anautogenous mosquitoes because they require a blood meal for the development and maturation of eggs [56,57]. In the last few decades, there has been emphasis on the study of regulatory pathways that are involved in vitellogenesis, the synthesis of YPPs, because of the prospect of identifying novel high-value targets for mosquito population control. There has been significant pursuit and progress in our understanding of the mechanisms that regulate vitellogenesis in mosquitoes, particularly in the yellow fever mosquito *A. aegypti* [58,59]. 

**Figure 5:**
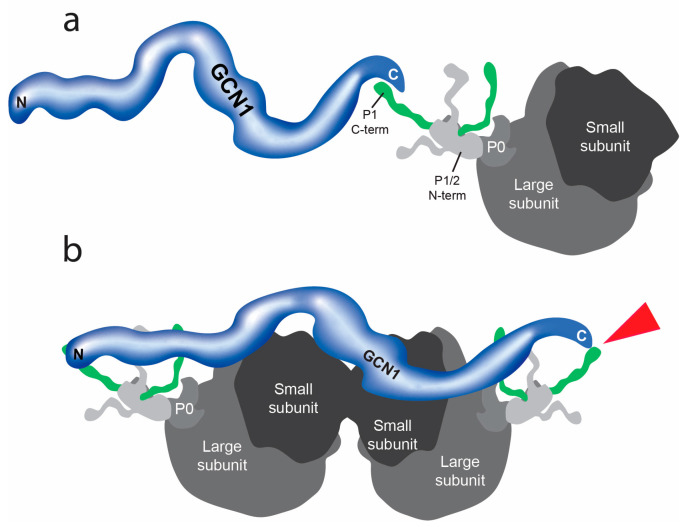
Schematic of the interaction of GCN1 with ribosomal protein P1. (**a**) The C-terminal of GCN1 interacts with ribosomal protein P1. Proteins P2 and P0 are part of the ‘stalk’ region of the larger subunit of eukaryotic ribosomes. (**b**) Hypothetical model of GCN1/P1 interactions with a colliding ribosome (left) and leading ribosome (right). The red arrowhead points to the protein/protein interaction between GCN1 and P1.

Earlier studies from our laboratory and others have investigated the mTOR and GAAC signaling pathways and their role in the regulation of mosquito vitellogenesis (see Figure 1c,e) [58,59,60]. A crucial upstream component of these pathways is cationic amino acid transporters (CATs). These transporters facilitate the movement of essential amino acids between midgut and fat body tissues during vitellogenesis and are hypothesized to be transceptors—functioning as both an amino acid transporter and receptor [57]. A recent Y2H screen using the C-terminus of CAT1 identified a protein–protein interaction with GCN1 [35]. In the present study, we performed a follow-up Y2H screen for potential protein–protein interactors of the C-terminus of GCN1 and identified an interaction with the 60S acidic ribosomal protein P1 (P1) (see Figure 5a). P1 is a component of the P-stalk region on the large subunit of eukaryotic ribosomes and has been shown to play an important role during the elongation stage of protein synthesis [61,62,63]. 

In eukaryotes, the P-stalk is made up of three acidic ribosomal proteins, P0, P1, and P2, which together form a pentameric P-complex with a ratio of 1:2:2 [64,65]. During translation, P1 and P2 are responsible for binding translation factors [66,67,68]. P1 and P2 proteins are present in the cytoplasm in both ribosome-bound and free forms. The exchange between these two pools regulates the activity of ribosomes [69]. 

Interestingly, it has been shown that in vitro, the presence of isolated P-stalks is sufficient to trigger GCN2-mediated phosphorylation of the eukaryotic initiation factor—2α (eIF2α) even in the absence of the rest of the ribosome [39]. We have shown in an earlier study that eIF2α is phosphorylated after a blood meal in the mosquito fat body [35].

Under amino acid starvation conditions or in the presence of other cellular stressors, ribosomes collide during mRNA translation [70,71,72]. A recently published cryo-electron microscopy study revealed the structure of GCN1 bound to two colliding ribosomes [73]. In this model, the N-terminus of GCN1 interacts with the P-stalk of the colliding ribosome while the C-terminus is closely located near the P-stalk of the leading ribosome. Considering the strong interaction between the C-terminus of GCN1 and P1 we found in the Y2H screen, we hypothesize that the N-terminal and C-terminal regions of GCN1 bind to the P-stalks of both the leading and the colliding ribosomes during amino acid starvation (see Figure 5b). 

In an interesting finding, it has been shown that P1/P2 coupled to ribosomes is necessary for flaviviruses like dengue viruses (DENV) and yellow fever virus (YFV) to replicate [74]. The substantial reduction in early DENV protein accumulation caused by a P1/P2 knockdown suggests that these P-stalk proteins could be necessary for viral protein translation.

Our study showed that *P1* was expressed in all mosquito organs tested (see Figure 3a). This suggests that *P1* is a ‘housekeeping gene’ with a constant level of expression [75]. Therefore, *P1* has the potential to be used as a control gene in quantitative PCR for unfed adult *A. aegypti*. 

Our Western blot results concerning the protein expression of P1 in different mosquito tissues showed bands at the predicted sizes of mosquito P1 (15 kDa) and the mosquito P1/P2 complex (38 kDa). The Western blot shown in Figure 3b also includes several bands at higher molecular weights in unique patterns in different tissues. We speculate that these bands represent either larger protein complexes that involve P1 or cross-reaction of the antibody with other mosquito proteins. To further investigate our findings, we plan to raise alternative antibodies against specific mosquito epitopes of P1. 

The phenotypes we observed after an RNAi-mediated knockdown of P1 were severe. Knockdown females laid no eggs after ingesting a blood meal, and they had significantly lower survival rates. This decrease in survival time in P1-knockdown mosquitoes may be due to the disruption in protein synthesis after injection. This likely temporarily impacts wound healing from the injection and diminishes their immunological defenses against infection. Development of ovaries and eggs of *P1*-dsRNA-injected mosquitoes was arrested, and those eggs did not mature (see Figure 4a,c). Our previous study on the CAT1 and GCN1 interaction showed that GCN1 knockdown affected mosquito fecundity but did not affect vitellogenin expression [35]. Earlier knockdown studies of proteins associated with the mTOR signaling pathway and *A. aegypti* CATs often resulted in a reduction in egg numbers but never total sterility [57,59,60,76]. P1 knockdown also caused a significant reduction in the survival of female mosquitoes; in contrast, knockdown of GCN1 did not cause any mortality.

## 5. Conclusions

We identified P1 as an interactor of GCN1 in the mosquito fat body and found that it was necessary for mosquito egg development. *A. aegypti* females, when injected with *P1* dsRNA, lost their capacity to produce eggs after a blood meal. Our results show that *P1* is a housekeeping gene that is necessary for mosquito reproduction. 

## 6. Future Directions

Our results point towards P1 and other ribosomal stalk proteins as potential targets for the development of dsRNA-based mosquito birth control. The next steps for this novel research include investigating the duration of the ovary phenotype after P1 knockdown, studying the effects of knockdown of the other P-stalk proteins, testing for larvicidal activity of dsRNA against P1, and expanding our studies to other pest arthropods. 

## Figures and Tables

**Table 1:** dsRNA synthesis primers. T7 promoter regions are in bold.

Primer	Sequence	Product Size (bp)
*P1* forward	**TAATACGACTCACTATAGGGAG** ATGTTTTTCGTCATTGG	363
*P1* reverse	**TAATACGACTCACTATAGGGAG** TCAACCTGCGATTCC	
*GFP* forward	**TAATACGACTCACTATAGGG** CGATGCCACCT	518
*GFP* reverse	**TAATACGACTCACTATAGGG** CGGACTGGGTG	

**Table 2:** qRT-PCR primers.

Primer	Sequence	Product Size (bp)
*P1* forward	CGACGATGTCGCTGTGACCG	95
*P1* reverse	CCTTGACGAACAGAGCGGGC	
*β-actin* * forward	GACTACCTGATGAAGATCCTGAC	93
*β-actin* * reverse	GCACAGCTTCTCCTTAATGTCAC	

* *β-actin* = internal reference gene.

## Data Availability

The data presented in this study are available in the main text of the article.

## Supplementary Materials

The following supporting information can be downloaded at: https://www.mdpi.com/article/10.3390/insects15020084/s1, Figure S1: P1 expression in different mosquito organs and tissues tested (Figure 3b); Figure S2: Image for Actin control (Figure 3b); Figure S3: P1 expression western blot for different mosquito organs tested (Thorax, Fat Body, Mid Gut, Malpighian Tubules, Ovary, Head); Figure S4: Survival Curve Replicates (R1–R3), (Supplemental figure for Figure 3); Figure S5. Egg-laying paper with eggs and without eggs; Table S1. List of proteins identified after Yeast-Two Hybrid screen; Table S2: Mosquito survival data; Table S3. Measurements from mosquito ovaries post-injection and PBM.
