# Supplementary material for: RNAi-Mediated Knockdown of Acidic Ribosomal Stalk Protein P1 Arrests Egg Development in Adult Female Yellow Fever Mosquitoes, Aedes aegypti
Source: Insects. 2024 Jan 24;15(2):84. doi: 10.3390/insects15020084 (PMC10889338; doi:10.3390/insects15020084)
Supplement: Supplementary file 1 [file insects-15-00084-s001.zip › insects-2802290-supplementary.pdf]

Table S1. List of proteins identified after Yeast-Two Hybrid screen

S. NO.	Identified potential prey interactors	NCBI Accession
**1.**	PREDICTED: Aedes aegypti 60S acidic ribosomal protein P1 (LOC5565839), mRNA	XM_001650143.2
**2.**	PREDICTED: Aedes aegypti 60S acidic ribosomal protein P1-like (LOC110678133)	XM_021850740.1
**3.**	PREDICTED: Aedes aegypti 60S acidic ribosomal protein P1 (LOC5578368), mRNA	XM_001656889.2
**4.**	Aedes aegypti clone AE-294 60S acidic ribosomal protein P1 mRNA, complete cds	DQ440047.1
**5.**	PREDICTED: Aedes aegypti 60S acidic ribosomal protein P1 (LOC5577349), mRNA	XM_001656376.2
**6.**	PREDICTED: Aedes aegypti 60S acidic ribosomal protein P1 (LOC5578249), partial mRNA	XM_001663729.2

Figures S1- S3. The following page contains Western blot images used in main figures without cropping, color removal, and brightness/ contrast correction. The label above each image contains a reference to the main text figure where the image is included.

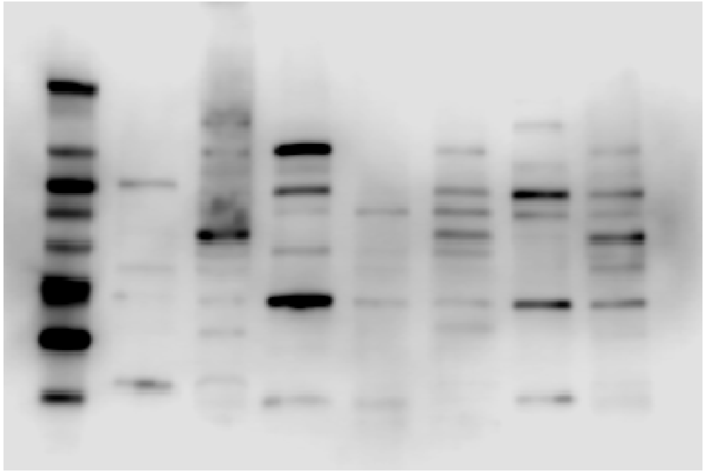

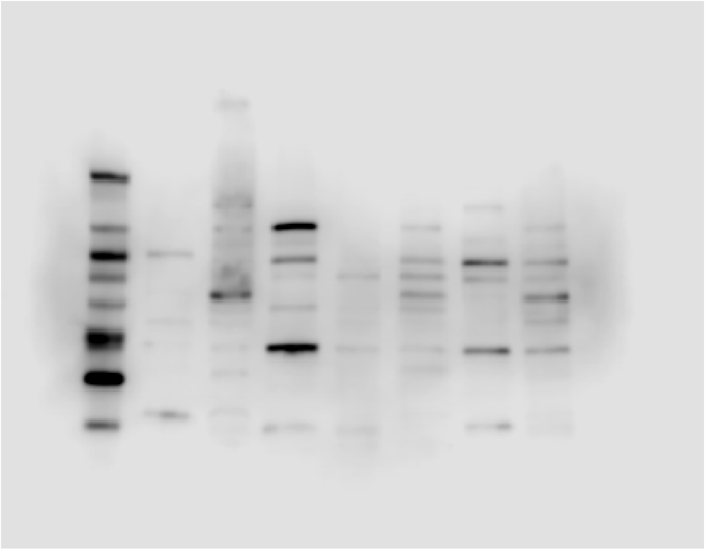

Figure S1. P1 expression in different mosquito organs and tissues tested (Fig. 3 b).

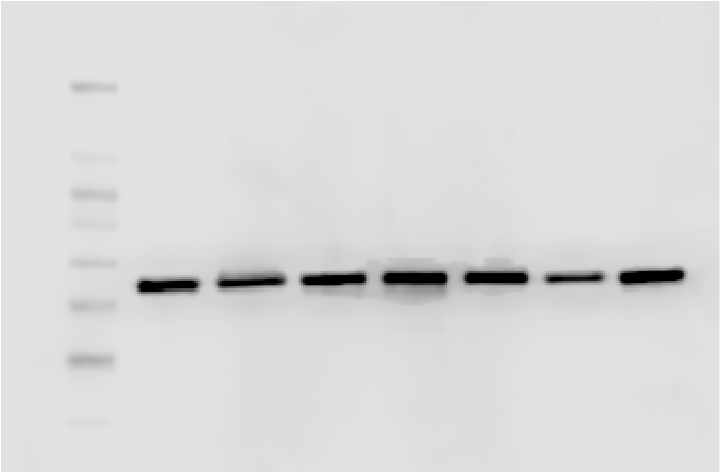

Figure S2. Image for Actin control (Fig. 3 b)

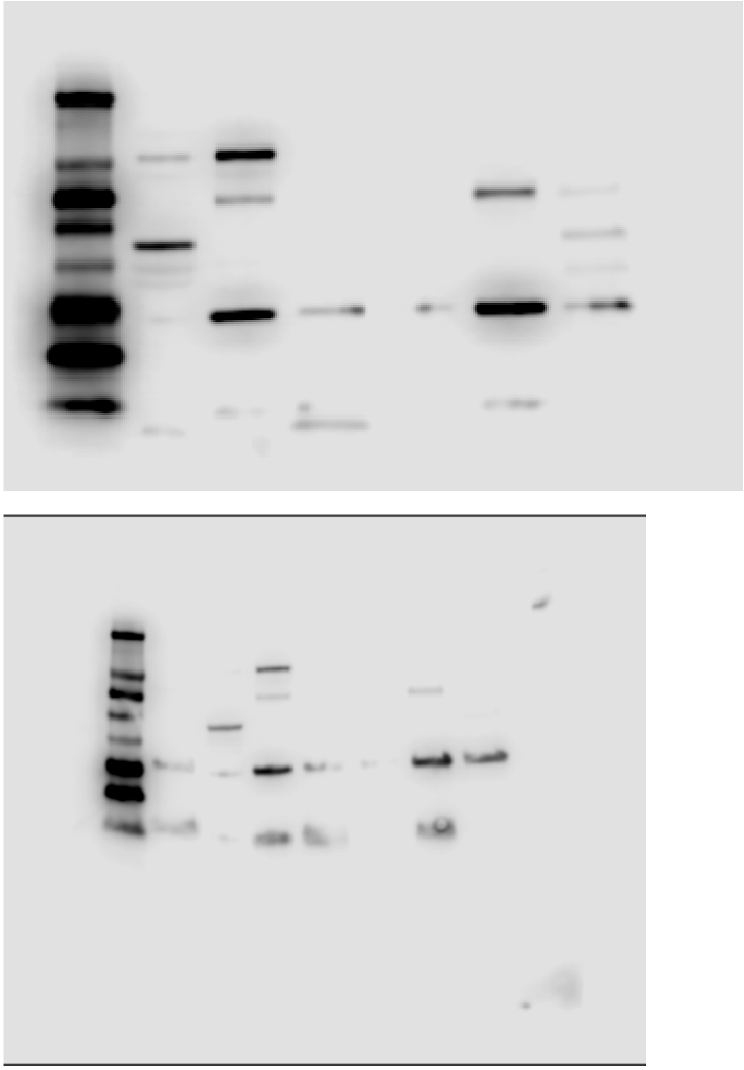

Figure S3. P1 expression western blot for different mosquito organs tested (Thorax, Fat Body, Mid Gut, Malpighian Tubules, Ovary, Head)

Table S2. Mosquito survival data

# mosquitoes dead (not cumulative)								
Time after dsRNA injection (hours)	GFP injected (control)			P1 injected (treatment)			AVG GFP injected (control)	AVG P1 injected (treatment)
	R1 (66 total)	R2 (68 total)	R3 (58 total)	R1 (66 total)	R2 (70 total)	R3 (60 total)		
0	0	0	0	0	0	0	0	0
24	2	7	1	5	19	4	3.33	9.33
48	1	4	2	2	10	5	2.33	5.66
72	7	3	2	16	8	7	4	10.33
96	1	2	0	3	5	3	1	3.66
# mosquitoes dead (cumulative)								
Time after dsRNA injection (hours)	GFP injected (control)			P1 injected (treatment)			AVG GFP injected (control)	AVG P1 injected (treatment)
	R1 (66 total)	R2 (68 total)	R3 (58 total)	R1 (66 total)	R2 (70 total)	R3 (60 total)		
0	0	0	0	0	0	0	0	0
24	2	7	1	5	19	4	3.33	9.33
48	3	11	3	7	29	9	5.66	15
72	10	14	5	23	37	16	9.66	25.33
96	11	16	5	26	42	19	10.66	29
# mosquiotes alive								
Time after dsRNA injection (hours)	GFP injected (control)			P1 injected (treatment)			AVG GFP injected (control)	AVG P1 injected (treatment)
	R1 (66 total)	R2 (68 total)	R3 (58 total)	R1 (66 total)	R2 (70 total)	R3 (60 total)		
0	66	68	58	66	70	60	64	65.33
24	64	61	57	61	51	56	60.66	56
48	63	57	55	59	41	51	58.33	50.33
72	56	54	53	43	33	44	54.33	40
96	55	52	53	40	28	41	53.33	36.33

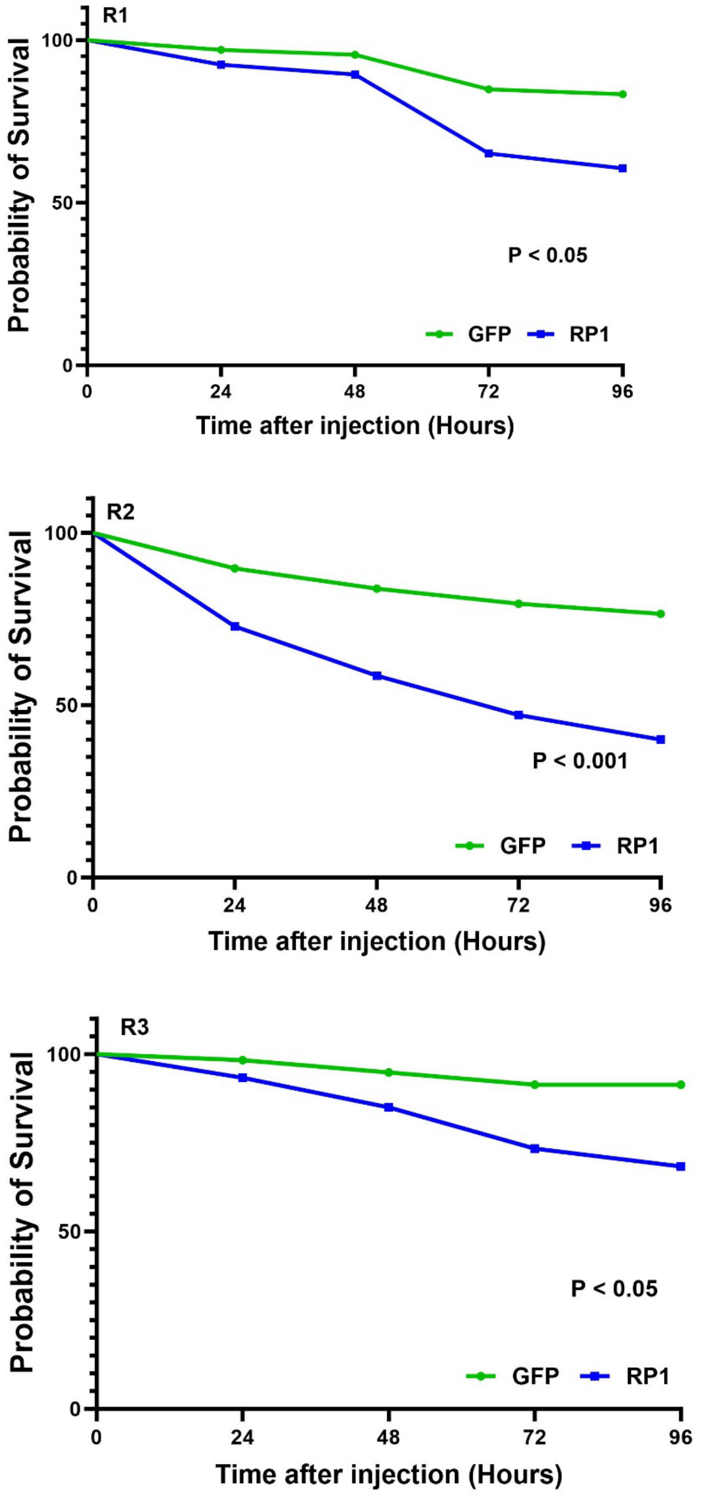

Figure S4. Survival Curve Replicates (R1-R3), (Supplemental figure for Figure 3.)

Table S3. Measurements from mosquito ovaries post-injection and PBM

Ovary length(mm)								
	**GFP**	**P1**	**GFP**	**P1**	**GFP**	**P1**	**GFP**	**P1**
	**0 Hr**	**0 Hr**	**24 Hr**	**24 Hr**	**48 Hr**	**48 Hr**	**72 Hr**	**72 Hr**
	0.9	0.7	2	1.3	2.8	1.4	3.1	1.6
	1	0.8	2.1	1.4	2.7	1.5	2.8	1.7
	0.7	0.7	1.9	1.3	2.8	1.4	3	1.4
	0.8	0.8	2.1	1.2	2.9	1.3	2.9	1.5
	0.9	0.8	2.1	1.3	3.1	1.7	2.8	1.3
	1	0.9	2	1.4	2.9	1.6	2.7	1.6
	0.8	0.8	2.1	1.5	2.8	1.4	2.8	1.4
	0.7	0.7	1.9	1.3	2.9	1.5	3	1.5
	0.8	0.8	2	1.3	2.7	1.3	2.6	1.3
	0.9	0.8	2.1	1.2	2.8	1.4	2.9	1.4
**Average**	**0.85**	**0.78**	**2.03**	**1.32**	**2.84**	**1.45**	**2.86**	**1.47**

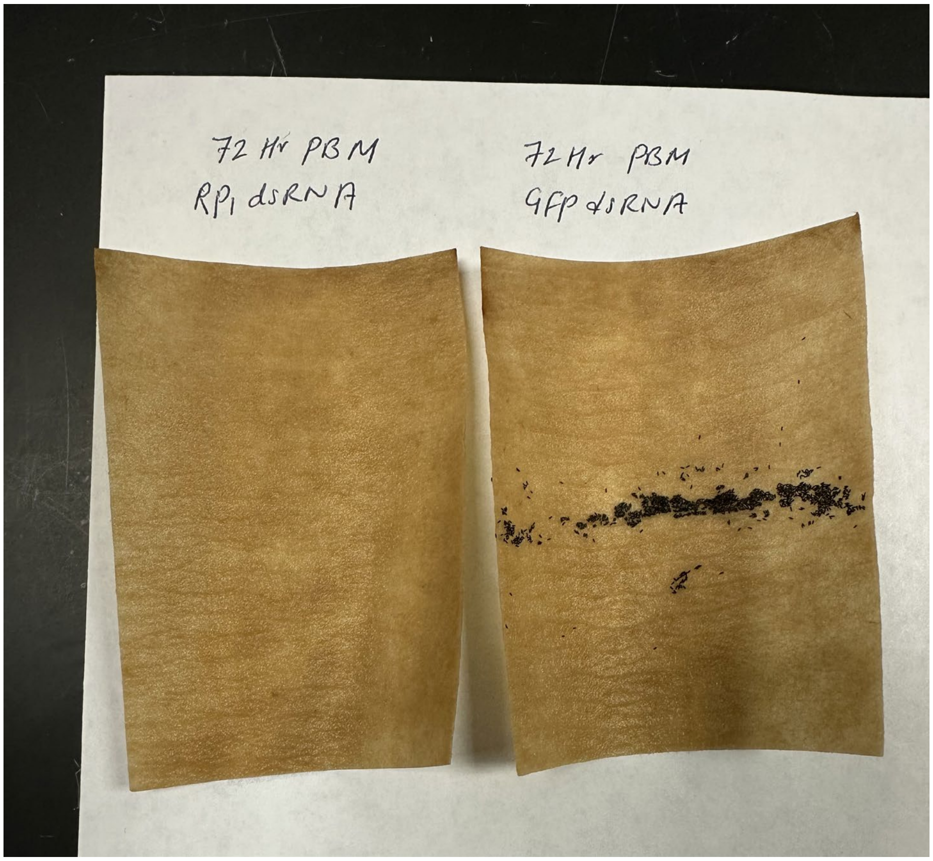

Figure S5. Egg-laying paper with eggs and without eggs
